# Varying VA intervals during Para‐Hisian pacing maneuver—What is the mechanism?

**DOI:** 10.1002/joa3.12650

**Published:** 2021-10-29

**Authors:** Debabrata Bera, Suchit Majumder, Sanjeev S. Mukherjee, Kuntal Bhattacharya

**Affiliations:** ^1^ Department Cardiology RTIICS Kolkata India; ^2^ Department of Cardiology Apollo Gleneagles Hospital Kolkata India; ^3^ Department of Cardiology Medica Super‐Speciality Kolkata India

**Keywords:** direct atrial capture, Para‐his pacing, short VA time, SVT maneuvers

## Abstract

Inadvertent direct atrial capture and pure his capture can result in variable findings during parahis pacing manoeuvre (PHP). Understanding the results and positioning the pacing bipole towards ventricular aspect (distal his region) is helpful to avoid ambiguous results during PHP.

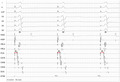

## 1 CASE

**FIGURE 1:**
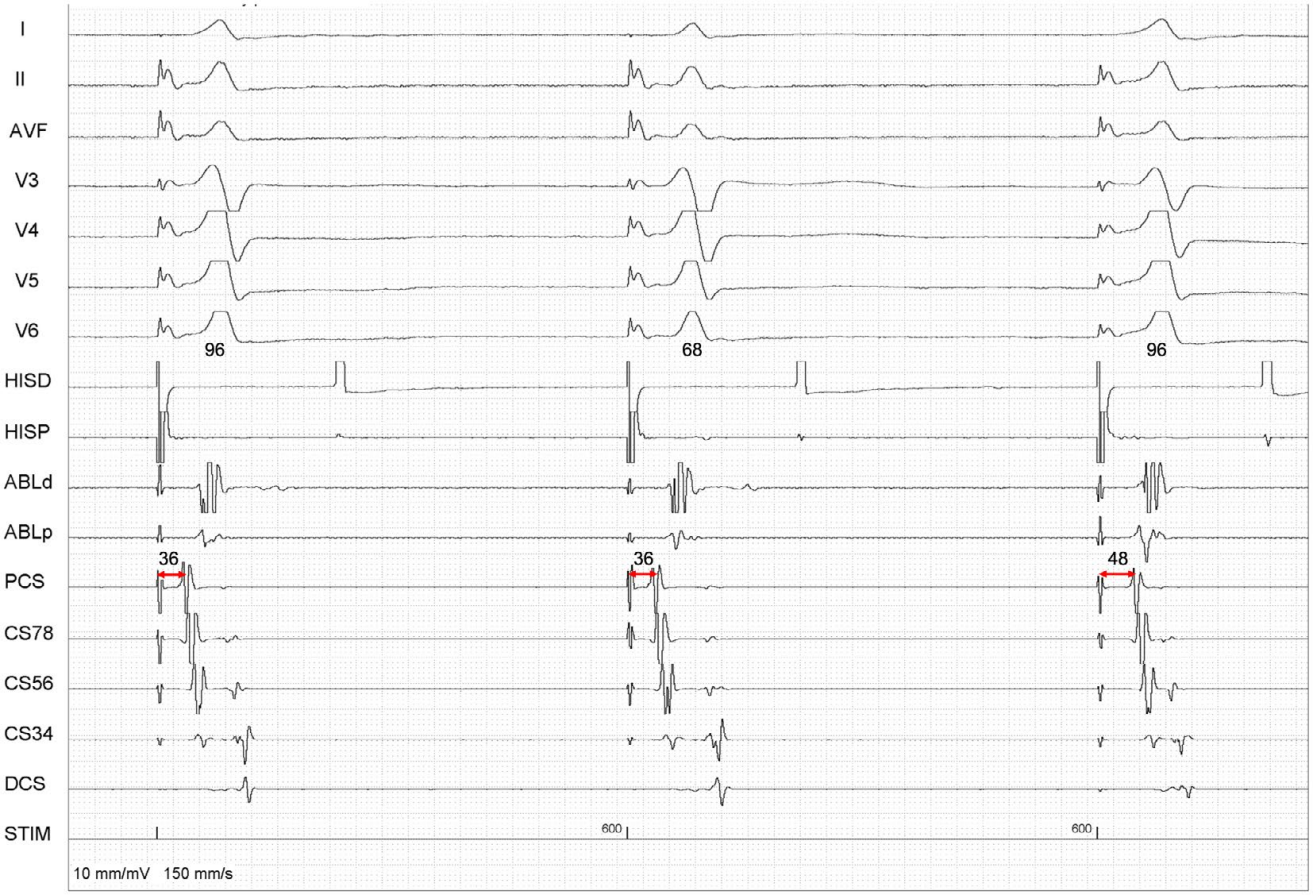
The Para‐Hisian pacing was performed from HisD bipole. The ABL catheter is in RV apex. The SA time is measured in proximal coronary sinus (CS) in msec. The second beat is narrower (68 ms) with an isoelectric segment suggestive of pure His capture, as compared to the first/third beat (width = 96 ms) with His plus myocardial capture. The third beat has a short VA time of 48 ms masquerading as direct atrial capture. But when compared to shorter SA (36 ms) of first two beats [and longer SA subsequently described in Figure 2, SA of 140 ms] this SA (of 48 ms) was proven to be resulting from a true VA conduction

**FIGURE 2:**
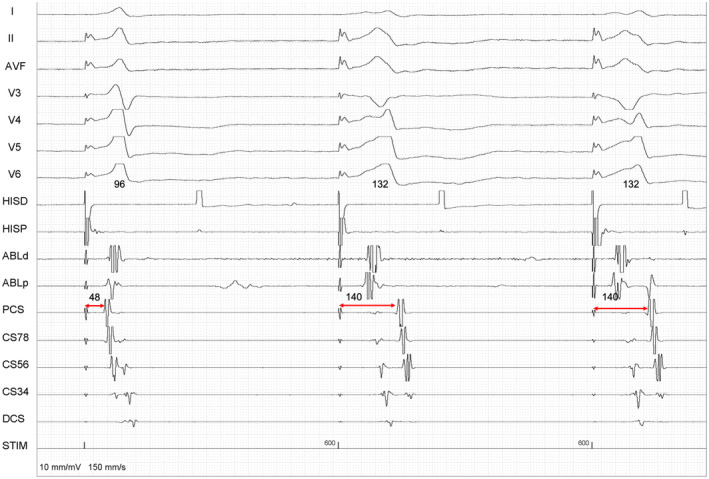
The second and third beats are notched and wider (132 ms) suggestive of only myocardial capture. The VA time prolongs by 92 ms in wider beat confirming a nodal response. This retrospectively supports that the third beat in Figure 1 was nodal conduction as compared to DAC in first two beats. The 92 ms prolongation is speculated to a concomitant retrograde RBBB

A 50‐year‐old man with a structurally normal heart underwent an electrophysiology study (EPS) for recurrent supraventricular tachycardia. Baseline ECG did not have any preexcitation. His VA conduction was concentric and decremental. There was no VA jump. During EPS Para‐Hisian pacing (PHP) maneuver was performed as a routine protocol at pacing cycle length (PCL) of 600 ms with decremental output. What are the findings in Figures 1 and 2 and what is the route of VA conduction?

## 2 DISCUSSION

Although His signal (H) release is the ideal way to analyze His capture during PHP, in the index case it was decided on the basis of QRS morphology as H signal was not well discernible. There are three QRS complexes in Figure 1. At first glance, it appears that the very short SA (Stimulus‐A EGM time) is due to direct atrial capture (DAC) in all three beats (Figure 1). But on measurements, it is noted that the third beat has a SA/VA time longer (48 ms) than the first two beats (36 ms). It is also noted that the first and third QRS complexes resulted from para‐his capture (H + V). In contrast, the second beat is a selective His capture (H) with an isoelectric segment and late “V‐EGM” (along with inadvertent DAC). The same can be evident from 12 lead ECG (Appendix 1). Unless the pure His capture and DAC are recognized a false interpretation of extra‐nodal response could be made from the first two beats with fixed SA. Therefore, in Figure 1, there are two different SA times, despite His capture in all three beats. The first beat with SA time of 36 ms which is certainly DAC. The reasons for SA increment could be—(a) release of DAC (DAC to true VA conduction) or (b) SA increase due to intra‐atrial delay (unlikely in absence of PCL change).

Figure 2 proves that the VA conduction is via AV node. During isolated myocardial (V) capture in the wider second beat‐ the SA time is prolonged by 92 ms (48‐140 ms). However, such a long increment (92 ms) cannot be attributed to just a nodal VA conduction when H is not captured in the last two beats. There must be a retrograde RBBB that cannot be ascertained here in absence of another dedicated catheter in His/RB (right bundle) region. The other possibility could be a jump from a fast pathway (FP) to a slow pathway (SP), but in that case, the atrial activation pattern would have changed. Moreover, in absence of shortening of the HH interval, a block in FP is unlikely. Accessory pathway conduction (extranodal response) conduction is ruled out from this finding in Figure 2.

To complete the case, it turned out to be a case of slow‐fast AVNRT. He had antegrade AH jump and dual AV nodal physiology as well. VA jump could not be elicited though. He underwent successful SP modification.

These illustrative images during a PHP highlight that there can be exceptions to the rule that very short VA (<60 ms in proximal CS) is a marker for inadvertent DAC.^1^ Occasionally true VA during His capture can be very short masquerading as DAC when supra‐his conduction is brisk.^2^ It is also interesting to note that even during *true VA conduction* with His capture (third beat in Figure 1 and first beat in Figure 2), the A‐EGM can paradoxically precede the local V‐EGM (distinctly noted here in CS 34 and CS 56). This is because during His capture, the local VA in CS catheter is not a result of an activation in series, rather it is an activation in parallel, analogous to an AVNRT circuit. In contrast, during isolated myocardial capture (second beat), the local V has to precede local A‐EGM when VA activation takes place in series.^3^, ^4^

## CONFLICT OF INTEREST

None.

## Supporting information

Supplementary Material
