# Supplementary figures and images for: Varying VA intervals during Para‐Hisian pacing maneuver—What is the mechanism?
Source: J Arrhythm. 2021 Oct 29;37(6):1585–7. doi: 10.1002/joa3.12650 (PMC8637088; doi:10.1002/joa3.12650)

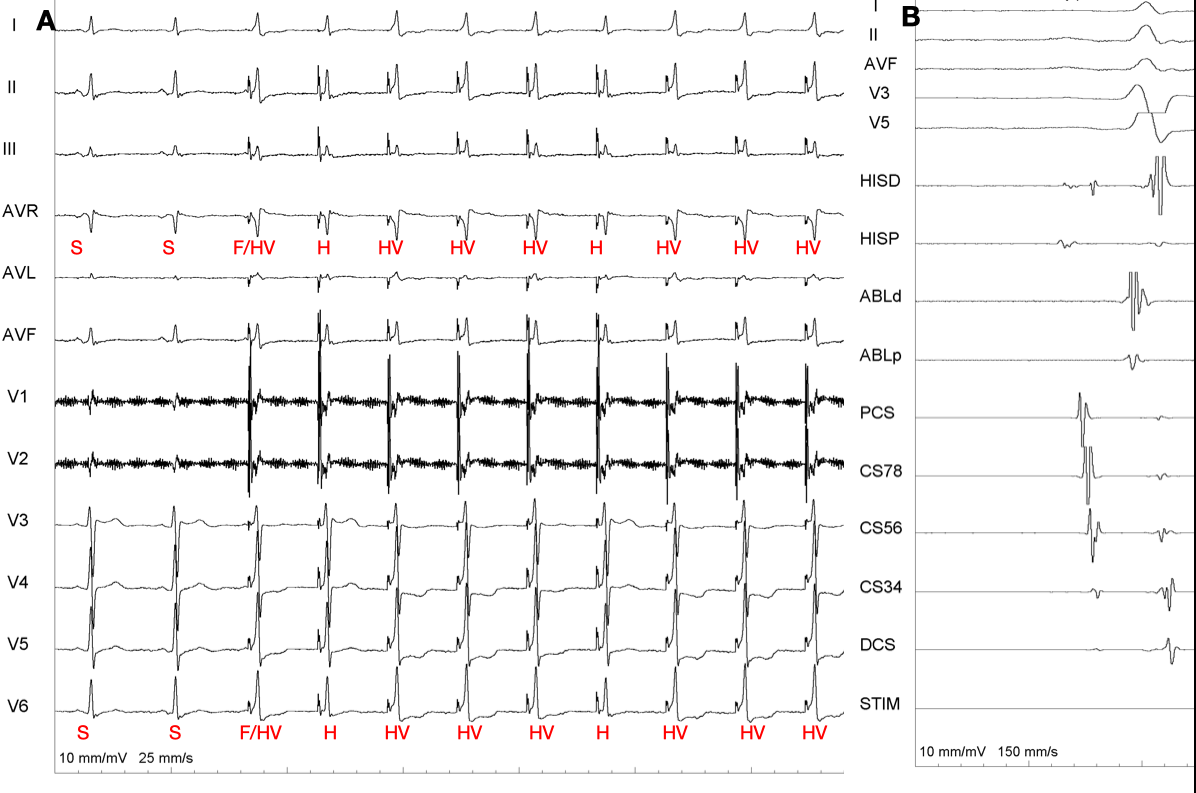

Supplement: Supplementary file 1 — Supplementary Material [file JOA3-37-1585-s001.png]
